# Perinatal Mortality and Morbidity of Growth Restricted Fetuses and Newborns (own Experience) – First Report

**DOI:** 10.34763/devperiodmed.20172101.2934

**Published:** 2017-05-29

**Authors:** Katarzyna Pankiewicz, Tomasz Maciejewski

**Affiliations:** 1 Department of Obstetrics and Gynecology, Institute of Mother and Child, Warsaw Poland

**Keywords:** fetal growth restriction, perinatal mortality and morbidity, wewnątrzmaciczne ograniczenie wzrastania płodu, umieralność i zachorowalność okołoporodowa płodów i noworodków

## Abstract

**Aim:**

to evaluate the outcome of pregnancies complicated by fetal growth restriction with particular emphasis on the factors (fetal and maternal) related to perinatal mortality and morbidity of the fetus and newborn.

**Material and methods:**

Retrospective analysis of the documentation of 53 women admitted with the diagnosis of fetal growth restriction based on ultrasound examination (fetal biometry and fetal vessel Doppler abnormalities). 38 (71.7%) patients were referred to our department with the diagnosis of fetal growth restriction, whereas 15 (28.3%) cases were diagnosed in our hospital. 32 (60.4%) women were referred to our department by their main obstetrician, 13 (24.5%) by other hospitals and 8 (15.1%) came to triage because of worrisome symptoms. The patients were divided into 2 groups according to the presence of fetal/neonatal complications: the first group (n=14) - with complications (defined as one or more of the following: stillbirth, neonatal death, respiratory distress syndrome (RDS), intraventricular haemorrhage (IVH) Grade III or IV , necrotic enterocolitis (NEC), proven neonatal sepsis or bronchopulmonary dysplasia (BPD)) and the second one (n=39) – without severe complications.

**Results:**

Gestational age at diagnosis and at delivery was lower in the first group (28.5 weeks vs. 32.15 weeks, p=0.003 and 29.2 weeks vs. 32.8 weeks, p=0.0004). Female fetuses predominated in the second group (64.1%), whereas male fetuses in the first group (64.3%). In both groups the majority of patients delivered by cesarean section (92.9% vs. 97.4% p=0.44). Birth weight was significantly lower in the first group (774g vs. 1416g, p<0.0001). Perinatal morbidity (severe neonatal complications) occurred in 14 (26.4%) cases. The fetal and newborn perinatal mortality rate in the studied group was 13.19% (in comparison to 0.6% for the entire population of pregnant women in Poland).

**Conclusions:**

1. Gestational age (at diagnosis and at delivery) and birth weight are the most important prognostic factors related to the adverse outcome in the management of fetal growth restriction. 2.The most common mode of delivery for fetuses with growth restriction is the cesarean section. 3. Early detection of fetal growth restriction in routine perinatal care seems to be insufficient. 4. Fetal and newborn perinatal mortality and morbidity rates in fetal growth restriction are still high and the management of such pregnancies should take place in reference obstetric units, where detailed diagnostics, monitoring and treatment of fetal and neonatal complications can be performed.

## Streszczenie

**Celem pracy:**

była ocena wyników ciąż powikłanych wewnątrzmacicznym ograniczeniem wzrastania płodu ze szczególnym uwzględnieniem czynników (matczynych i płodowych) związanych z umieralnością i zachorowalnością okołoporodową płodów i noworodków.

**Materiał i metody:**

Analiza retrospektywna dokumentacji 53 pacjentek przyjętych do Kliniki Położnictwa i Ginekologii z rozpoznaniem wewnątrzmacicznego ograniczenia wzrastania płodu na podstawie badania ultrasonograficznego (biometria oraz nieprawidłowości w badaniu dopplerowskim przepływów w naczyniach płodowych). 38 (71,7%) kobiet zostało skierowanych do naszego ośrodka z rozpoznaniem FGR, natomiast w 15 (28,3%) przypadkach FGR rozpoznano w trakcie pobytu w Klinice. 32 (60,4%) ciężarnych zostało skierowanych przez lekarza prowadzącego, 13 (24,5%) z innego szpitala, a 8 (15,1%) zgłosiło się do Izby Przyjęć z powodu niepokojących objawów. W zależności od występowania powikłań płodowych i noworodkowych pacjentki podzielono na dwie grupy: pierwsza (n=14) – z powikłaniami (definiowanymi jako jedno lub więcej z następujących: wewnątrzmaciczne obumarcie płodu, śmierć noworodka, zespół zaburzeń oddychania, wylewy dokomorowe st. III lub IV, martwicze zapalenie jelit, posocznica i dysplazja oskrzelowo-płucna) , druga (n=39) – bez powikłań.

**Wyniki:**

Wiek ciążowy w momencie rozpoznania i w momencie porodu był istotnie niższy w pierwszej grupie (28,5 tygodni vs 32,15 tygodni, p=0,003 i 29,2 tygodni vs 32,8 tygodni, p=0,0004). W grupie z ciężkimi powikłaniami noworodkowymi przeważały płody płci męskiej (64,3%). W obu grupach najczęstszą drogą porodu było cięcie cesarskie (92,9% vs 97,4% p=0,44). Masa urodzeniowa noworodka była istotnie mniejsza w grupie pierwszej (774 g vs 1416 g, p<0,0001). Ciężkie powikłania noworodkowe wystąpiły w 14 (26,4%) przypadkach. Wskaźnik umieralności okołoporodowej płodów i noworodków w grupie badanej wynosił 13,19% (w porównaniu do 0,6% dla ogólnej populacji ciężarnych w Polsce).

**Wnioski:**

1.Wiek ciążowy w momencie rozpoznania i przy porodzie oraz masa urodzeniowa noworodka są najważniejszymi czynnikami prognostycznymi niekorzystnego wyniku położniczego w przypadku wewnątrzmacicznego ograniczenia wzrastania płodu. 2. Najczęstszą drogą porodu w przypadku wewnątrzmacicznego ograniczenia wzrostu płodu jest cięcie cesarskie. 3. Wczesne wykrywanie wewnątrzmacicznego ograniczenia wzrastania płodu w rutynowej opiece perinatalnej wydaje się być niewystarczające. 4. Wskaźniki umieralności i zachorowalności okołoporodowej płodów i noworodków w przypadku wewnątrzmacicznego ograniczenia wzrostu płodu są nadal wysokie, dlatego opieka nad ciężarnymi z tym powikłaniem powinna odbywać się w ośrodkach referencyjnych, w których możliwa jest właściwa diagnostyka, monitorowanie powikłań i leczenie noworodka.

## Introduction

Fetal growth restriction (FGR) is nowadays one of the most important problems in maternal-fetal medicine. It is associated with an increased risk for perinatal complications, such as fetal hypoxemia, acidemia, low Apgar scores and intrapartum distress. Perinatal mortality and morbidity rates are even ten times greater than those in normal age-matched controls [1]. Intrauterine growth restriction is also known as the single largest risk factor for stillbirth [2]. The management of FGR is thus a crucial dilemma, because it boils down to balancing between iatrogenic prematurity and stillbirth due to terminal placental dysfunction.

## Aim of the study

The aim of the study was to evaluate the outcome of pregnancies complicated by fetal growth restriction with particular emphasis on the factors (maternal and fetal) related to perinatal mortality and morbidity of the fetus and newborn.

## Material and methods

Retrospective analysis of the documentation of 53 women admitted to the Department of Obstetrics and Gynecology of the Institute of Mother and Child in Warsaw between 2009 and June 2014 with the diagnosis of FGR. Patients included in the study group were women with a singleton fetus at 25 to 36 weeks’ gestation. FGR was defined as fetal abdominal circumference (AC) < 10^th^ percentile and umbilical artery Doppler pulsatility index > 95^th^ percentile.

38 (71.7%) patients were referred to our department with the diagnosis of fetal growth restriction, whereas in 15 (28.3%) cases intrauterine growth restriction was diagnosed during ultrasound examination performed in our hospital. 32 (60.4%) women were referred to our department by their main obstetrician, 13 (24.5%) patients were admitted from other hospitals ( with fewer references) and 8 (15.1%) gravids came to triage because of worrisome symptoms (such as high blood pressure, abdominal pain, bleeding).

Multiple pregnancies and fetuses with antenatally diagnosed chromosomal aberrations or congenital abnormalities were excluded from the study. Each patient was monitored by cardiotocography and Doppler ultrasonography.

We evaluated the following parameters:

1. Maternal:
  – Medical history and course of pregnancy before admission to the clinic on the basis of medical records (such as pregnancy card, previous ultrasound examinations)
  – Gestational age at diagnosis and delivery
  – Coexistence of hypertensive disorders: gestational hypertension (GH), preeclampsia (PE) and preexisting hypertension (PEH)
  – The presence of oligohydramnion
  – Mode of delivery (vaginal or cesarean section)
  – Indications for delivery (fetal and maternal)
2. Fetal and neonatal:
  – Ultrasound AC and estimated fetal weight on diagnosis
  – Fetal vessel Doppler ultrasonography
  – Birth weight
  – Apgar scores in the 1^st^ and 5^th^ minute
  – Severe neonatal complications (adverse outcome) defined as: stillbirth, neonatal death, respiratory distress syndrome (RDS), intraventricular haemorrhage (IVH) Grade III or IV, necrotic enterocolitis (NEC), proven neonatal sepsis or bronchopulmonary dysplasia (BPD).

Depending on the presence of fetal or neonatal complications patients were divided into 2 groups: the 1st group (n=14) − with severe complications defined as above and the 2nd group (n=39) – without severe complications.

Fetal perinatal morbidity was defined as the presence of one or more of the following neonatal complications: respiratory distress syndrome (RDS), intraventricular haemorrhage (IVH) Grade III or IV , necrotic enterocolitis (NEC), proven neonatal sepsis or bronchopulmonary dysplasia (BPD).

Fetal perinatal mortality was defined as the number of fetal deaths and early neonatal deaths and expressed as a percentage of total births in the studied group. Fetal death (stillbirth) was the death of a fetus from 22 weeks’ gestation. Early neonatal death was the death in the first 7 days of life of a liveborn newborn.

Statistical analysis of collected data were performed using the statistical so$ware package PQStat ver. 1.4.8. Patients from both study groups were compared using the U Mann-Whitney test depending on the character of distribution. Categorical variables were compared with the Chi squared test with Yates correction and Fisher’s exact test. P values less than 0.05 were considered statistically significant.

## Results

There was no significant di&erence between the groups according to the patient’s age, parity, coexistence of hypertensive disorders and the presence of oligohydramnion (see table I). The mean gestational age at FGR diagnosis was 31 weeks. The gestational age at diagnosis was lower in the group with adverse outcome (28.5 weeks vs. 32.15 weeks, p=0.003) − see table I. Female fetuses predominated in the group without severe neonatal complications (64.1%), whereas male fetuses in the group where adverse outcome occurred (64.3%). This di&erence was statistically significant (p=0.03). Intrauterine fetal death in the studied group occurred in 1 case (1.89%).

**Table I:** Maternal characteristics. Tabela I. Charakterystyka matek.

	No adverse outcome (without severe morbidity) *Bez niekorzystnego wyniku położniczego* n=39	Adverse outcome^*^ (severe morbidity) *Niekorzystny wynik położniczy* n=14	p
Maternal ** age (**years) *Wiek matki (lata)*	31.8±3.64	31.2±4.9	0.53
Nulliparity *pierworódki*	27 (69.2%)	10 (71.4%)	0.88
Preeclampsia *Stan przedrzucawkowy*	1 (2.6%)	2 (14.6%)	0.94
Gesta**tional hypertension *Nadciśnienie ciążowe*	20 (51.3%)	5 (35.7%)	0.32
** Pre-existing ** hypertension *Nadciśnienie istniejące uprzednio*	1 (2.6%)	2 (14.6%)	0.10
Oligohydramnion *Małowodzie*	12 (30.8%)	5 (35.7%)	0.44
Gestational age at FGR diagnosis^ (weeks) *Wiek ciążowy w momencie rozpoznania FGR (tygodnie)*	32.15±2.65	28.5±2.88	**<0.05 (0.0003)**

* adverse outcome(severe morbidity) = the presence of one or more of the following neonatal complications: respiratory distress syndrome, intraventricular haemorrhage (IVH) Grade III or IV , necrotic enterocolitis (NEC), proven neonatal sepsis or bronchopulmonary dysplasia (BPD). Niekorzystny wynik położniczy = jedno lub więcej z następujących powikłań: wewnątrzmaciczne obumarcie płodu, śmierć noworodka, zespół zaburzeń oddychania, wylewy dokomorowe st. III lub IV, martwicze zapalenie jelit (NEC), posocznica i dysplazja oskrzelowopłucna **Bolded** values are statistically significant **Wytłuszczone** są wartości istotne statystycznie

The most common indications for delivery were fetal causes. In the group with severe neonatal complications abnormal fetal vessel Doppler indices (most of all: umbilical artery absent/reversed end-diastolic * ow) were detected more o$en, whereas in the group without adverse outcome abnormal non-stress cardiotocography was more common. However, this difference was not statistically significant (see table II).

**Table II:** Indications for delivery and mode of delivery. Tabela II. Wskazania do porodu i droga porodu.

	No adverse outcome (without severe morbidity) *Bez niekorzystnego wyniku położniczego* n=39	Adverse outcome ^*^ (severe morbidity) *Niekorzystny wynik położniczy n=14*	P
Gestational age at delivery (weeks)^ *Wiek ciążowy przy porodzie (tygodnie)*	32.8±2.64	29.2±3.02	**<0.05 (0.0004)**
Indications for delivery: *Wskazania do porodu* Fetal *płodowe* Maternal *matczyne*	37(94.9%) 5 (12.8%)	13(92.9%) 1(7.1%)	0.78 0.56
Cesarean section *Cięcie cesarskie*	38 (97.4%)	13 (92.9%)	0.44
** Vaginal delivery *Poród pochwowy*	1 (2.6%)	1 (7.1%)	0.96

* adverse outcome(severe morbidity) = the presence of one or more of the following neonatal complications: respiratory distress syndrome, intraventricular haemorrhage (IVH) Grade III or IV , necrotic enterocolitis (NEC), proven neonatal sepsis or bronchopulmonary dysplasia (BPD). Niekorzystny wynik położniczy = jedno lub więcej z następujących powikłań: wewnątrzmaciczne obumarcie płodu, śmierć noworodka, zespół zaburzeń oddychania, wylewy dokomorowe st. III lub IV, martwicze zapalenie jelit (NEC), posocznica i dysplazja oskrzelowopłucna **Bolded** values are statistically significant **Wytłuszczone** są wartości istotne statystycznie

The gestational age at delivery was lower in the group with severe fetal/neonatal complications (29.2 weeks vs. 32.8 weeks, p=0.0004). In both groups the majority of patients delivered by cesarean section (92.9% vs. 97.4% p=0.44) (tab. II).

52 out of 53 (98%) babies were liveborn, but 6 of them (11.5%) died. Newborn birth weight was significantly lower in the group with severe complications than in the second group (774 g vs 1416 g, p<0.0001). The 1^st^ minute Apgar score was substantially lower in the group with severe neonatal complications than in the second group (4.4 vs 7.8, p=0.0003). However, there was no difference between the groups in the 5^th^ minute Apgar score evaluation (p=0.83) (tab. III).

**Table III:** Neonatal characteristics. Tabela III. Charakterystyka noworodków.

	No adverse outcome (without severe morbidity) *Bez niekorzystnego wyniku położniczego n=39*	Adverse outcome ^*^ (severe morbidity) *Niekorzystny wynik położniczy n=14*	p
** Birth weight (grams)^ ** *Masa urodzeniowa (gramy)*	1416±402	774±447	**<0.05 (<0.0001)**
Infant ** male gender *Płeć męska*	14 (35.9%)	9 (64.3%)	**<0.05 (0.048)**
1^st^ minute Apgar score *Punktacja Apgar w 1 minucie*	7.8±1.7	4.3±3	**<0.05 (0.0003)**
** 5^th^ minute ** Apgar ** score *Punktacja Apgar w 5 minucie*	8.7±1.1	8±2.4	0.84

* adverse outcome(severe morbidity) = the presence of one or more of the following neonatal complications: respiratory distress syndrome, intraventricular haemorrhage (IVH) Grade III or IV , necrotic enterocolitis (NEC), proven neonatal sepsis or bronchopulmonary dysplasia (BPD). Niekorzystny wynik położniczy = jedno lub więcej z następujących powikłań: wewnątrzmaciczne obumarcie płodu, śmierć noworodka, zespół zaburzeń oddychania, wylewy dokomorowe st. III lub IV, martwicze zapalenie jelit (NEC), posocznica i dysplazja oskrzelowopłucna **Bolded** values are statistically significant **Wytłuszczone** są wartości istotne statystycznie

The fetal and newborn perinatal mortality rate for the entire study group was 13.19%. Neonatal morbidity concerned 14 (26.4%) cases. That means 74.6% babies survived without severe complications.

## Discussion

The fetal and newborn perinatal mortality rate in the fetal growth restriction group (13.19% in our study) is substantially higher than for the entire population of pregnant women (in Poland it is about 0.6%). Only 71.7% of the patients were referred to the hospital with the diagnosis of fetal growth restriction, whereas 28.3% women were diagnosed already in our department. Moreover, only slightly more than half of the patients were diagnosed by their main obstetricians. That shows that early detection of intrauterine growth restriction in routine perinatal care may be insufficient.

The fetal and neonatal perinatal mortality rate shown in our study is higher than those presented in the two largest and most important European FGR trials. In the GRIT study, the perinatal mortality rate was about 10%, in the TRUFFLE study it was about 8% [3, 4]. This is probably related with the relatively small number of patients in our research in comparison with these two large studies. However, there is a study of 180 babies with birth weight < 10^th^ percentile, <34 weeks and with abnormal umbilical Doppler, delivered between 1997-2004 in one of the units later included in the TRUFFLE group. The overall mortality rate in that study was 14% and the severe morbidity rate 28.3%. The definition of severe morbidity was similar, but included retinopathy of prematurity [5].

The rate of babies who met the criteria of the composite outcome of death or severe morbidity was lower than in the TRUFFLE study. The only case of stillbirth in our research was caused by a decision for non-intervention, because the prognosis was considered too poor.

It is known that gestational age and birth weight are some of the most important factors in the prediction of severe morbidity and mortality in fetal growth restriction [3, 6]. Our study confirmed that gestational age − both at diagnosis and at delivery - was lower in the group with adverse outcome, while birth weight was almost two times lower than in the group of babies who survived without severe morbidity. It has also been revealed in earlier studies that female gender is a positive prognostic factor in cases of extremely low birth weight [7. 8]. In our research the majority of the fetuses without severe complications were female as well.

The most common mode of delivery was the cesarean section. Other studies presented similar results. Because of significant placental dysfunction, fetuses with growth restriction are at very high risk of acidemia, intrapartum distress and central nervous system injury, so for the majority of them vaginal delivery can increase this risk and the C-section seems to be safer [1, 9].

It is interesting to note the lack of relationship between severe neonatal complications and the mother’s hypertensive disorders. Preeclampsia was diagnosed in 21% of the patients (20.5% patients in the first group and 21.4% patients in the second group). This rate is significantly higher than in the entire population of pregnant women, where preeclampsia a&ects 5-8% of all pregnancies [10]. The coexistence of FGR and preeclampsia results from the very similar pathomechanism of these two conditions. They are both related to abnormal trophoblast invasion to maternal spiral arteries and in consequence placental dysfunction. There is not enough evidence to explain why some patients develop preeclampsia, while others develop FGR. It is probably connected with many other factors, especially with genetic and immunological conditions [11]. On the other hand, the coexistence of FGR and preeclampsia should be related with higher risk of severe morbidity. In the TRUFFLE study the presence of gestational hypertension at study entry was one of the most important independent determinants of death or severe morbidity [3]. In our research there was no statistically significant di&erence between the groups according to any hypertensive disorder (GH, PE and PEH). To explain it, we must emphasize that our study was a retrospective analysis and in some cases pregnancy-induced hypertension or preeclampsia developed during the time between FGR diagnosis and delivery. Probably, the reason for a similar preeclampsia rate in both groups can be the appearance of late onset preeclampsia in the group without adverse outcome. It is known that the risk of complications bound to late onset preeclampsia is not so high as in the case of early onset disease [1, 6]. Pre-existing hypertension in our study occurred more o$en in the group with death or severe morbidity (14.3% vs. 2.6%), but this difference was not statistically significant either (p=0.167). It can be related with the relatively small number of patients with this condition in both groups.

In summary – the main limitation of this study is the low number of patients. However, it is the first report and we are going to present results of a significantly greater group of patients in the future.

## Conclusions

1. Gestational age at diagnosis, gestational age at delivery and birth weight are the most important prognostic factors related to the adverse outcome in the management of fetal growth restriction.
2. The most common mode of delivery for fetuses with growth restriction is the cesarean section.
3. Early detection of fetal growth restriction in routine perinatal care seems to be insufficient.
4. Fetal and newborn perinatal mortality and morbidity rates in fetal growth restriction are still high and the management of such pregnancies is a big challenge, so it should take place in reference obstetric units, where detailed diagnostics, monitoring and treatment of fetal and neonatal complications can be performed.
